# Supplementary material for: Motivation and physical activity across Chinese adolescents: Based on latent profile analysis
Source: PLoS One. 2025 Jul 30;20(7):e0328383. doi: 10.1371/journal.pone.0328383 (PMC12310012; doi:10.1371/journal.pone.0328383)

PLOS ONE

Motivation and Physical Activity across Chinese Adolescents: Based on Latent Profile Analysis

--Manuscript Draft--

**Manuscript Number:**	PONE-D-25-04270
**Article Type:**	Research Article
**Full Title:**	Motivation and Physical Activity across Chinese Adolescents:<br><br>Based on Latent Profile Analysis
**Short Title:**	Motivation and Physical Activity across Chinese Adolescents
**Corresponding Author:**	Wei Zheng, Ph.D<br>Sanming University<br>Sanming City, Fujian Province CHINA
**Keywords:**	Motivation, physical activity, latent profile analysis, adolescent
**Abstract:**	Objectives: Using the person-centered approach, this paper explores the relationship between adolescent motivation and physical activity. We aim to identify the latent motivation profiles and examine their difference in physical activity.<br>Methods: A sample of 571 Chinese adolescents (M age = 11.995, SD = 1.519) were analyzed in this study.<br>Results: The results of latent profile analysis indicate that three profile model is the optimal model and the subgroup with higher scores of intrinsic motivation and external motivation reported more physical activity. Moreover, the physical activity of male adolescents is significantly more active than that of female adolescents, while older adolescents have less physical activity than younger adolescents.<br>Conclusion: This study identified adolescents with different motivation profiles and physical activity. Findings suggest the need for more personalized strategies to promote adolescent participation in physical activity and provide a novel insight into intervention for adolescents with low motivation. Further research could be measured by objective methods and long-term design.
**Order of Authors:**	Ying Zhao, Ph.D
	Qinghua Wu
	Wei Zheng, Ph.D
**Additional Information:**	
**Question**	**Response**
**Financial Disclosure**<br><br>Enter a financial disclosure statement that describes the sources of funding for the work included in this submission. Review the submission guidelines for detailed requirements. View published research articles from *PLOS ONE* for specific examples.<br><br>This statement is required for submission and **will appear in the published article** if the submission is accepted. Please make sure it is accurate.	The author(s) received no specific funding for this work.

Powered by Editorial Manager® and ProduXion Manager® from Aries Systems Corporation

**Funded studies**<br>Enter a statement with the following details:<br>• Initials of the authors who received each award<br>• Grant numbers awarded to each author<br>• The full name of each funder<br>• URL of each funder website<br>• Did the sponsors or funders play any role in the study design, data collection and analysis, decision to publish, or preparation of the manuscript?<br><br>Did you receive funding for this work?	
**Competing Interests**<br><br>Use the instructions below to enter a competing interest statement for this submission. On behalf of all authors, disclose any competing interests that could be perceived to bias this work—acknowledging all financial support and any other relevant financial or non-financial competing interests.<br><br>This statement is required for submission and **will appear in the published article** if the submission is accepted. Please make sure it is accurate and that any funding sources listed in your Funding Information later in the submission form are also declared in your Financial Disclosure statement.<br><br>View published research articles from *PLOS ONE* for specific examples.	NO authors have competing interests

Powered by Editorial Manager® and ProduXion Manager® from Aries Systems Corporation

**NO authors have competing interests**<br><br>Enter: *The authors have declared that no competing interests exist.*<br><br>**Authors with competing interests**<br><br>Enter competing interest details beginning with this statement:<br><br>*I have read the journal's policy and the authors of this manuscript have the following competing interests: [insert competing interests here]*<br><br>* typeset	
**Ethics Statement**<br><br>Enter an ethics statement for this submission. This statement is required if the study involved:<br><br>• Human participants<br>• Human specimens or tissue<br>• Vertebrate animals or cephalopods<br>• Vertebrate embryos or tissues<br>• Field research<br><br>Write "N/A" if the submission does not require an ethics statement.<br><br>General guidance is provided below. Consult the submission guidelines for detailed instructions. **Make sure that all information entered here is included in the Methods section of the manuscript.**	This research was approved the written consent by the institutional ethical committee of the Guangzhou Sport University (No. 2025LCLL-009).

**Format for specific study types**<br><br>**Human Subject Research (involving human participants and/or tissue)**<br>• Give the name of the institutional review board or ethics committee that approved the study<br>• Include the approval number and/or a statement indicating approval of this research<br>• Indicate the form of consent obtained (written/oral) or the reason that consent was not obtained (e.g. the data were analyzed anonymously)<br><br>**Animal Research (involving vertebrate animals, embryos or tissues)**<br>• Provide the name of the Institutional Animal Care and Use Committee (IACUC) or other relevant ethics board that reviewed the study protocol, and indicate whether they approved this research or granted a formal waiver of ethical approval<br>• Include an approval number if one was obtained<br>• If the study involved *non-human primates*, add *additional details* about animal welfare and steps taken to ameliorate suffering<br>• If anesthesia, euthanasia, or any kind of animal sacrifice is part of the study, include briefly which substances and/or methods were applied<br><br>**Field Research**<br><br>Include the following details if this study involves the collection of plant, animal, or other materials from a natural setting:<br>• Field permit number<br>• Name of the institution or relevant body that granted permission	
**Data Availability**<br><br>Authors are required to make all data underlying the findings described fully available, without restriction, and from the time of publication. PLOS allows rare exceptions to address legal and ethical concerns. See the PLOS Data Policy and FAQ for detailed information.	Yes - all data are fully available without restriction

A Data Availability Statement describing where the data can be found is required at submission. Your answers to this question constitute the Data Availability Statement and **will be published in the article**, if accepted.<br><br>**Important:** Stating 'data available on request from the author' is not sufficient. If your data are only available upon request, select 'No' for the first question and explain your exceptional situation in the text box.<br><br>Do the authors confirm that all data underlying the findings described in their manuscript are fully available without restriction?	
**Describe where the data may be found in full sentences. If you are copying our sample text, replace any instances of XXX with the appropriate details.**<br><br>• If the data are **held or will be held in a public repository**, include URLs, accession numbers or DOIs. If this information will only be available after acceptance, indicate this by ticking the box below. For example: *All XXX files are available from the XXX database (accession number(s) XXX, XXX.*).<br>• If the data are all contained **within the manuscript and/or Supporting Information files**, enter the following: *All relevant data are within the manuscript and its Supporting Information files.*<br>• If neither of these applies but you are able to provide **details of access elsewhere**, with or without limitations, please do so. For example:<br><br>*Data cannot be shared publicly because of [XXX]. Data are available from the XXX Institutional Data Access / Ethics Committee (contact via XXX) for researchers who meet the criteria for access to confidential data.*<br><br>*The data underlying the results presented in the study are available from (include the name of the third party*	The data for this study can be obtained by contacting the corresponding author (zhengweiSanmingUniversity@outlook.com).

and contact information or URL).<br>• This text is appropriate if the data are owned by a third party and authors do not have permission to share the data.<br><br>* typeset	
Additional data availability information:	

Manuscript

Click here to access/download;Manuscript;Motivation and physical activity across Chinese adolescent.docx

Motivation and Physical Activity across Chinese Adolescents: Based on Latent Profile Analysis

Ying Zhao[1] Qinghua Wu[2] Wei Zheng[3]*

1. School of Physical Education and Health Care, Sanming University

2. Graduate school, Guangzhou Sport University

3. School of Physical Education and Health Care, Sanming University

Author Note

Ying Zhao https://orcid.org/0009-0009-1996-6992

Qinghua Wu https://orcid.org/0009-0002-9648-5630

Wei Zheng* https://orcid.org/0009-0006-4711-8986

Wei Zheng is a professor at the School of Physical Education and Health Care, Sanming University, interested in physical education and training.

Correspondence concerning this article should be addressed to Wei Zhen*, School of Physical Education and Health Care, Sanming University, No. 25 Jingdong Road, Sanming City, Fujian Province, China, Post Code: 365004.

Email: zhengweiSanmingUniversity@outlook.com

Ethics statement

The studies involving humans were approved by the Human Experimental Ethics Inspection of Guangzhou Sport University (No. 2025LCLL-009). The studies were conducted by the local legislation and institutional requirements.

Author contributions

Ying Zhao:Conceptualization, Formal analysis, Writing-original draft.

Qinghua Wu: Data collecting

Wei Zheng: Methodology, Writing – review & editing, Funding acquisition.

Motivation and Physical Activity across Chinese Adolescents:

Based on Latent Profile Analysis

Abstract

Objectives: Using the person-centered approach, this paper explores the relationship between adolescent motivation and physical activity. We aim to identify the latent motivation profiles and examine their difference in physical activity.

Methods: A sample of 571 Chinese adolescents (*M* age = 11.995, *SD* = 1.519) were analyzed in this study.

Results: The results of latent profile analysis indicate that three profile model is the optimal model and the subgroup with higher scores of intrinsic motivation and external motivation reported more physical activity. Moreover, the physical activity of male adolescents is significantly more active than that of female adolescents, while older adolescents have less physical activity than younger adolescents.

Conclusion: This study identified adolescents with different motivation profiles and physical activity. Findings suggest the need for more personalized strategies to promote adolescent participation in physical activity and provide a novel insight into intervention for adolescents with low motivation. Further research could be measured by objective methods and long-term design.

Keywords: Motivation, physical activity, latent profile analysis, adolescent

Introduction

Moderate physical activity (PA) has a positive impact on the health of adolescents, a recommendation from WHO that children and adolescents should spend 60 minutes on moderate-vigorous physical activity (MVPA) per day (Chaput et al., 2020). However, about 80% of adolescents lack daily PA (van Sluijs et al., 2021) and Insufficient PA may lead to diseases (cardiometabolic and mental health disorders) and obesity. This issue has also attracted widespread attention in China (Yan et al., 2024; Zhu et al., 2017).

Therefore, the purpose of this study was to explore the relationship between motivation and PA among adolescents. In particular, the present study used the person-centered approach to explore the latent profiles of motivation and examine the differences in PA among each subgroup. Results can provide a novel insight into intervention for PA among adolescents.

Motivation and Physical Activity

PA can positively influence people's physical health (Jago et al., 2023; White et al., 2017). Especially, PA is especially beneficial for weight control, the development of the cardiovascular system, and cancer prevention (Warburton & Bredin, 2017). In addition, PA also has positive effects on people's mental health, such as promoting psychological health and cognitive abilities (Eather et al., 2023). Therefore, WHO and many countries are recommending adolescents engage in PA and maintain this habit (Y. Liu et al., 2016), which is beneficial for their health and academic achievement.

Unfortunately, adolescents are relatively inactive in many countries (Guthold et al., 2010). Early studies found that lacking PA is a growing problem among children and adolescents in China (Lu et al., 2017), which has a negative impact on the health of teenagers (L. Liu et al., 2024). Although the Chinese government has tried various ways to increase PA among students (Gao et al., 2024), there are still many students who are in a state of insufficient physical activity. In particular, male students preferred PA to female students (Y. Liu et al., 2016, 2023) and younger students' engagement in sports was more frequent than older students (Xiang et al., 2023). This phenomenon was underpinned by various key variables, which needed to be identified.

Relative research suggests that both external and internal factors can affect adolescents' engagement with PA. External factors, such as parents and peers, are exactly vital for students' PA. For example, parental encouragement and support positively relate to children and adolescent PA (Biddle et al., 2011). Their parents mainly initiate children's sports participation at the beginning and parental support is necessary for children and adolescents during participation (Burke et al., 2024; Doggui et al., 2021). Although parental influence is important to children, this influence will shift to classmates when children get older (Eccles, 1999), supporting that the influence from peers can affect young adolescents' PA engagement effectively (Pluta et al., 2020). For example, peer support can promote adolescent participation in sports (Chen et al., 2017).

Internally, according to social cognitive theory, self-efficacy is the ability and

capability perceived by individuals to perform their behavior (Bandura, 1977, 1997). Self-efficacy is tightly related to health behavior, such as PA (Dominick et al., 2013), students with high self-efficacy have higher participation in sports activities (Williams & Rhodes, 2016). Moreover, passion can also influence people's intention and participation in sports (Paradis et al., 2013), people with harmonious passion prefer to maintain their exercise habits (Rodrigues & Teixeira, 2023).

Based on the Self-Determination Theory (SDT), previous studies have shown that motivation is vital for PA (Owen et al., 2014). Motivation can be divided into intrinsic motivation, external motivation, and amotivation (R. Ryan & Deci, 2000b). In particular, intrinsic motivation is defined as the engagement of activity for its inherent satisfaction (R. Ryan & Deci, 2000a), which can be divided into three types. Firstly, intrinsic motivation to know relates to engagement for the satisfaction of learning, exploration, and understanding. Secondly, intrinsic motivation toward accomplishments is defined as engagement for unique accomplishments. Finally, intrinsic motivation to experience stimulation is people’s engagement in their stimulating feelings (Pelletier et al., 1995).

Additionally, external motivation refers to doing something because of external value or regulation and external motivation can be divided into three types. Initially, external regulation is that people’s behavior is controlled by external resources. Furthermore, with introjection, people to some extent agree with external perspectives, and their behavior is not needed to initiate anymore. In addition, in the next stage, identification refers to people performing with internal regulation and

self-determination, although they are still motivated by extrinsic reasons (Pelletier et al., 1995). Unlike intrinsic and external motivation, amotivation is when people believe something has no value so they either do not act at all or act without intention (R. Ryan & Deci, 2000b).

Motivation is a key factor in PA. Early studies identified a positive relationship between motivation and adolescents' PA (Crane & Temple, 2015; Sterdt et al., 2014), adolescents with higher motivation for exercise are more likely to maintain good exercise habits. For example, an American study found that adolescents' motivations are positively associated with MVPA, and this relationship is moderated by settings (Nogg et al., 2021). Another Chinese study has shown the positive effects of intrinsic motivation on PA among Chinese students, which emphasized the importance of intrinsic motivation (Y. Liu et al., 2023).

However, motivation can positively promote PA but relatively research examines the influence of different types of motivations at the same time. Moreover, although many Chinese scholars focused on the relationship between motivation and PA based on the variable-centered, relatively less scholars explored the latent profile of motivation among adolescents by a person-centered approach, which can offer a novel sight for intervention in physical activity.

Variable-Centered Approach and Person-Centered Approach

The variable-centered approach is popular in social science, which aims to explore the relationship between variables. This method assumes that people in the

same sample are a set of “averaged” parameters (Morin et al., 2016). However, the person-centered approach provides another perspective to examine the assumption of people homogeneity (Howard & Hoffman, 2018).

The person-centered approach assumes that the sample could be divided into several subpopulations by different sets of parameters (Morin et al., 2016), which is becoming popular. This approach aims to identify the latent subgroup with a common feature based on a specific subset of variables (Howard & Hoffman, 2018). The strength of this approach is that it can provide finer-grained detail about the sample (Collins & Lanza, 2009) therefore it is suitable for research that aims to categorize latent subgroups and explore the differences of subgroups.

The person-centered approach can be divided into latent class analysis (LCA), latent profiles analysis (LPA), and so on. LCA is used for discrete observed variables, whereas LPA is used for continuous observed variables. LPA has received lots of attention and interest recently (Spurk et al., 2020) and is practical in the area of physical activity (D. Brown et al., 2020; Chen et al., 2024). For example, a study based on Spanish handball players identified distinctive profiles of motivation, fear, and anxiety and examined the differences between the profiles by covariables and specific playing positions (Gómez-López et al., 2021). In addition to athletes, some scholars also used latent profile analysis to explore students' motivation for physical activity. Haerens’s study investigated the relationship between college students’ physical motivation by latent profile analysis and results found that students with more optimal motivational profiles reported more engagement in PA (Haerens et al.,

2010). Another study identified latent motivational profiles for exercise among adults (Lindwall et al., 2017).

Compared with Western research, there are not many related studies in China and fewer scholars explore the latent profile in the motivation of PA, which can provide detailed information from a person-centered perspective for PA among adolescents.

The Current Research

This study examined the latent profiles of motivation among Chinese students and explored the difference in subgroups in physical activity by LPA. Therefore, this study assumes that (a) different categories of motivation are present in students and (b) there may be a significant difference in PA in different latent categories.

Methods

Participants

This research was approved by the institutional ethical committee of the university (No. 2025LCLL-009), students, school, their parents or guardians. Under the guidance of the teacher, students are required to complete the questionnaire within 20 minutes between 18th January, 2025 and 20th January, 2025. A total of 641 students were recruited from one primary school (5th and 6th) and one junior high school (7th) in the south of China and all students received a small gift after they finished the survey. We did not include a questionnaire that was blank or gave the same response (Krosnick, 1999) and the final sample was 571 (*M* age = 11.995, *SD* =

1.519, Age range = 10 to 14, Male = 279, Female = 296, Primary school student = 344, Junior high school student = 277). Based on past research, samples of 300 to 500 would qualify in latent profile analysis (Ferguson et al., 2020; Tein et al., 2013), and samples of 500 can lead to enough accuracy (Spurk et al., 2020), this research satisfied the requirements of sample size.

Measures

Motivation

Motivation was measured by the Chinese version of the Sports Motivation Scale, which was modified from the Sport Motivation Scale (F. Li & Harmer, 1996; Pelletier et al., 1995). The Chinese version of the Sports Motivation Scale consists of 7 dimensions: intrinsic motivation to know (4 items, such as for the pleasure it gives me to know more about the sport that I practice, α = 0.718), intrinsic motivation to accomplishment (4 items, α = 0.764), intrinsic motivation to experience stimulation (4 items, α = 0.762), introjection (4 items, α = 0.707), identification (4 items, α = 0.708), external regulation (4 items, α = 0.741) and amotivation (4 items, α = 0.720). The scale used a 5-point Likert scale, where 1 indicates "Not agree at all" and 5 indicates "Very strongly agree". This scale has demonstrated good applicability in Chinese research (Qurban et al., 2019).

Physical Activity

Physical activity was assessed metabolic equivalent of task (MET) of the student and measured by the International Physical Activity Questionnaire short form

(IPAQ-SF), which consists of 7 items (Craig et al., 2003). Six items investigate the participants' physical activity, and one item examines the duration of sedentary behavior. The weekly MET for each participant is calculated as the sum of the activity type value × weekly frequency × daily duration. Especially, MET in this research was calculated as 8.8 (vigorous-intensity activity) × weekly frequency × daily duration + 4.0 (moderate-intensity activity) × weekly frequency × daily duration + 3.3 (moderate-intensity) × weekly frequency × daily duration (Esmaeilzadeh et al., 2022; Fan et al., 2014). IPAQ-SF is considered effective and recommended in many countries (Rodríguez-Muñoz et al., 2017; Terwee et al., 2010). In this study, the calculated MET values are standardized before further analysis, and the extreme values (less than -3.29 or more than +3.29) will be eliminated (Osborne, 2012).

Demographic Variables

Demographic variables included students' gender (male = 1, female = 2), age, and education (primary school student = 1, junior high school student = 2).

Statistical Analysis

Descriptive statistics, correlations, analysis of variance (ANOVA), and multivariate analysis of variance (MANOVA) were analyzed by SPSS 25.0. Moreover, this research used Mplus 8.3 to conduct an LPA of the scores on the seven dimensions of sports motivation. Results of the Shapiro-Wilk test showed that all dimensions were non-normally distributed so this research used robust maximum likelihood estimation for analysis (Satorra, 1994).

This research examined 1-6 profiles to explore the optimal profile based on the recommendation of model fit (Ferguson et al., 2020). The optimal profile model is determined by AIC, BIC, aBIC, LMR, BLRT, and Entropy (Spurk et al., 2020). The smaller values of AIC, BIC, and aBIC indicate the better model fit. Additionally, if the values of BLRT and LMR are significant, the model fit of the *k* category is significantly better than the *k-1* category. Finally, entropy represents the accuracy of the model category and the values of 0.8 or greater suggest the high accuracy of the model category (Ferguson et al., 2020; Tein et al., 2013).

Results

Multicollinearity and Common Methods Bias

Multicollinearity represents a high degree of linear intercorrelation between explanatory variables and leads to incorrect results of regression analyses (Kim, 2019) and this research used variance inflation factor (VIF) to examine the multicollinearity. The results of VIF were 1.156-3.411 (Table 1), which showed that there was no severe multicollinearity in this research.

Table 1
Results of VIF

	Variables	Tolerance	VIF
1	Intrinsic Motivation to Know	0.405	2.469
2	Intrinsic Motivation to Accomplishment	0.322	3.104
3	Intrinsic Motivation to Experience Stimulation	0.294	3.411
4	Introjected Regulation	0.360	2.779
5	Identified Regulation	0.424	2.361
6	External Regulation	0.417	2.396
7	Amotivation	0.865	1.156

Common method bias (CMB) is a potential problem in behavior research (P. Podsakoff et al., 2003) and may cause a negative impact on the relationships between constructs (P. M. Podsakoff et al., 2024). This research used Harman's single-factor test to measure the CMB and the result showed account for the variance in the variables is 35.351%, which illustrated that this research did not have a substantial amount of common method variance.

Descriptive Statistics and Correlation Analysis

The results of descriptive statistics and Pearson's correlation analysis are shown in Table 2. Intrinsic motivation to know was positively correlated with intrinsic motivation to accomplishment (r = .667, p < .001), intrinsic motivation to experience stimulation (r = .737, p < .001), introjection (r = .662, p < .001), identification (r = .585, p < .001), external regulation (r = .542, p < .001), and physical activity (r = .156, p < .001). Intrinsic motivation to accomplishment was positively correlated with intrinsic motivation to experience stimulation (r = .757, p < .001), introjection (r = .725, p < .001), identification (r = .643, p < .001), external regulation (r = .648, p < .001), and physical activity (r = .152, p < .001). Intrinsic motivation to experience stimulation was positively correlated with introjection (r = .715, p < .001), identification (r = .636, p < .001), external regulation (r = .660, p < .001), and physical activity (r = .172, p < .001). Introjection was positively correlated with identification (r = .626, p < .001), external regulation (r = .666, p < .001) and physical activity (r = .159, p < .001). Identification was positively correlated with external

regulation (r = .668, p < .001), amotivation (r = .224, p < .001), and physical activity (r = .106, p = .012). External regulation was positively correlated with amotivation (r = .226, p < .001), and physical activity (r = .133, p < .001).

However, amotivation was not correlated with intrinsic motivation to accomplishment (r = -.022, p = .610), intrinsic motivation to experience stimulation (r = .038, p = .365), introjection (r = .022, p =.606), identification (r = .081, p = .056), and physical activity (r = -.063, p = .134).

This is not according to the table

Table 2

Descriptive Statistics and Correlations of Key Variables

	Variables	*M*	*SD*	Ske	Hur	1	2	3	4	5	6	7
1	Intrinsic Motivation to Know	3.627	0.774	-0.738	1.145							
2	Intrinsic Motivation to Accomplishment	3.656	0.826	-0.816	1.135	.667***						
3	Intrinsic Motivation to Experience Stimulation	3.461	0.849	-0.502	0.399	.737***	.757***					
4	Introjection	3.575	0.785	-0.577	0.906	.662***	.725***	.715***				
5	Identification	3.443	0.751	-0.647	1.176	.585***	.643***	.636**	.626***			
6	External Regulation	3.432	0.825	-0.597	0.790	.542***	.648***	.660**	.666***	.668***		
7	Amotivation	2.917	0.882	-0.103	-0.344	-.022	.038	.022	.081	.244***	.226***	
8	Physical activity	-0.064	0.808	1.687	2.977	.156**	.152**	.172***	.159***	.106*	.133**	-.063

Notes. * p < 0.05,** p < 0.01, *** p < 0.001. Ske = Skewness, Kur = Kurtosis.

Latent Profile Analysis of Motivation

This research used LPA to divide motivation of PA into categories and the model fitting indices of 1 to 6 categories are shown in Table 4. The values of AIC, BIC, and a BIC were shapely decrease from 1 category to 3 categories whereas slowly

decreasing from 4 categories to 6 categories, indicating that the model with more categories was better. However, the *p*-value of the LMR of the 4-category model is more than .05 which indicating that the model with 3-category model was no significantly better than 3-category model. Finally, the value of entropy in 3-category model is more than 0.8, indicating the high accuracy of latent profile. Overall, category 3 could be considered as the optimal latent profile model according to the above parameters.

Table 3

Latent Profile Model Information Criteria, Likelihood Ratio Test, and Entropy (N = 571)

Model	*K*	Log(*L*)	AIC	BIC	aBIC	Entropy	BLRT	LMR	Group size
1	14	-4765.965	9559.930	9620.794	9576.350	/	/	/	1
2	22	-4199.404	8442.807	8538.450	8468.610	0.905	<0.001	0.0334	0.1821/0.8179
3	30	-3831.872	7723.744	7854.165	7758.929	0.886	<0.001	0.0019	0.0845/0.6061/0.3094
4	38	-3676.141	7428.282	7593.483	7472.850	0.860	<0.001	0.2273	0.0583/0.5131/0.2795/0.1492
5	46	-3591.447	7274.894	7474.874	7328.844	0.852	<0.001	0.1287	0.0366/0.0989/0.3609/0.3847/0.1189
6	54	-3533.188	7174.377	7409.136	7237.710	0.830	<0.001	0.5602	0.0337/0.0871/0.3321/0.3043/0.1347/0.1081

Additionally, the distribution of scores on the seven dimensions of the 3-category model is shown in Figure 1. Firstly, Profile 1 had the smallest number of students (8.45%), 48 students, and the lowest scores in intrinsic motivation and external motivation but their score of amotivation was relatively high than other dimensions. This result showed that this group of students was lack of intrinsic motivation and external motivation as well as not felt competent in physical activity. Therefore, this group was named as "low motivation group". Secondly, Profile 2, with a total of 345 students (60.61%), had moderate scores in intrinsic motivation and external motivation but their score of amotivation was the highest, so it was named "balanced motivation group". Finally, Profile 3 (30.94% and 178 students) scored significantly higher than other two group in intrinsic motivation and external motivation whereas

their score in amotivation was lower than Profile 3, which was named “high motivation group”.

Figure 1

Motivational Profiles of the Best Fitting Model (4 Profiles)

[Figure 1]

Moreover, this research conducted a covariate analysis (gender and education) in the 3-category model (Table 4). Results suggested gender was not significantly different between Profile 1 (Low Motivation Group) and Profile 2 (Moderate Motivation Group) whereas significantly different between Profile 1 and Profile 3 (Moderate Motivation Group), with more male students represented in the high motivation group. The coefficient of Profile 1 was bigger than Profile 3, indicating that the score of male motivation in Profile 3 was higher than Profile 1. For education, scores were also not statistically significantly different between Profile 1 and Profile 2 but significantly different for Profile 1 as compared to Profile 3. Negative coefficients indicated the scores of primary school students in the high-motivation group were higher than the low-motivation group.

Table 4

Covariate Analysis Results for Four-Profile Model

Variable	Group 2 (Moderate Motivation Group (n = 345)	Group 3 High Motivation Group (n = 136)
Gender	-0.134	-0.957**
Education	-0.205	-1.363**

Note. Group 1 (Low Motivation Group, n = 48) served as the reference group. ** $p < 0.01$.

Relationship between Covariate Variables and Physical Activity

MANOVA was used to compare the differences in gender and education in PA (Table 5). When gender is used as an independent variable, the scores of males' PA are significantly more than females (F (3, 364) = 4.714, p = .030). When age is used as an independent variable, there is no significant difference between primary school students and junior high school students (F (3, 364) = 1.799, p = .180), although the scores of primary school students (M = -0.0001, SD = 0.8253) were higher than junior high school students (M = -0.1570, SD = 0.7740).

Table 5

Results of MANOVA of Gender and Education

Variable	$M \pm SD$		F	p
	**Male**	**Female**		
Physical Activity	-0.0146 ± 0.8460	-0.1077 ± 0.7700	4.714	.030
	**Primary School Students**	**Middle School Students**		
Physical Activity	-0.0001 ± 0.8253	-0.1570 ± 0.7740	1.799	.180

Relationship between Motivational Profiles and Physical Activity

One-way ANOVA was used to compare the differences between 3 profiles in PA (Table 6) and results showed that differences across the 3 profiles (F (2, 565) = 12.523, p < .001). Profile 1, with the lowest score in physical activity, was significantly lower than Profile 3 (p < .001) whereas not significantly lower than Profile 3 (p = .405). Moreover, Profile 2 was significantly lower than Profile 3 (p < .001).

Table 6

Results of ANOVA of LPA

Variables	N	M	SD	Grade	p
Group 1<br>(Low Motivation Group)	48	-0.3091	0.7073	Group 2<br>Group 3	.405<br>< .001

Group 2 (Moderate Motivation Group)	345	-0.1526	0.7471	Group 3	< .001
Group 3 (High Motivation Group)	178	0.1759	0.8926		

Discussion

This study utilized LPA to explore potential categories of Chinese adolescent motivation, focusing on the differences among the subgroups in physical activity. The three latent profile model was the optimal profile model in this study. The scores of motivations in Profile 3 (High Motivation Group) were significantly higher than any other profiles and the physical activity of Profile 3 was also the highest according to the results of ANOVA.

Latent Categories of Motivation in Physical Activity

The results of LPA indicated that there were three subgroups of motivation in physical activity, namely, Profile 1 (Low Motivation Group), Profile 2 (Moderate Motivation Group), and Profile 3 (High Motivation Group).

Profile 1 accounts for 8.45%, whose scores in motivation are much lower than any other profile. In particular, the score of intrinsic motivation to experience stimulation in this subgroup is lower than any other dimensions and the score of amotivation is the highest, which means that adolescents in this subgroup lack motivation or intention for PA. Although this group has the smallest number of adolescents, it still requires the attention of parents and teachers and needs to improve sports motivation.

Profile 2 has the highest number of students (60.61%) and its scores are more even in each dimension except for amotivation and the score of intrinsic motivation to accomplishment is the highest. The result indicates that the intrinsic motivation and external motivation of adolescents in this subgroup are developed in a balanced way.

Additionally, the students of Profile 3, which is 30.94%, are less than Profile 2 and have the highest scores in intrinsic motivation and external motivation. The score of intrinsic motivation to accomplishment is the highest and the score of amotivation is much lower than other dimensions in this subgroup, which means that adolescents in this subgroup prefer to engage in PA.

Based on covariate analysis, there are no significant between Profile 1 and Profile 2 whereas there are significant differences between Profile 1 and Profile 3 both in gender and education. These results indicate that males are more active than females and primary school students are more active than junior high school students in motivation, consistent with previous findings. For example, a study based on Portuguese junior high school students showed that boys show higher motivation than girls and older students show lower motivation (Antunes et al., 2024). Another Chilean study found that male adolescents had statistically significantly more self-determined motivation toward exercise than female adolescents (Mayorga-Vega et al., 2019).

There may be several reasons for gender differences in motivation. Firstly, gender stereotypes can influence teenagers' choices and participation in sports. During childhood, children may develop stereotypes about sports influenced by their

environment (Chalabaev et al., 2013; Qu, 2023), such as those reinforced by social media or television viewing, which can lead to beliefs about gender limitations in certain domains (Davies et al., 2002). Moreover, the physiological differences between boys and girls can also end this difference to a certain extent (Eagly, 1995), boys' growth rate begins to accelerate during adolescence, gradually becoming stronger than girls in abilities such as strength and speed. This may make girls realize that boys may be more suitable for sports and weaken their motivation for physical activity.

Difference of Gender and Education in Physical Activity

Early studies used the variable-centered approach to explore the relationship between motivation and other variables about sports. Relatively fewer scholars in China focus on motivation and PA among adolescents from a person-centered perspective, which could provide some specific information.

The results of gender and education differences in PA were consistent with previous research, which has shown that male adolescents were more active than female adolescents and the PA of junior high school students was lower than that of elementary school students (Hong et al., 2020; Slater & Tiggemann, 2011; Xiang et al., 2023).

According to developmental theory, the reason why girls are less active than boys can be divided into peers and parents (Pluta et al., 2020). Girls are more likely to experience teasing from their peers during PA (Slater & Tiggemann, 2011), which

may negatively influence their flow and enjoyment (Vu et al., 2006) and decrease girls' sports engagement. Moreover, in addition to peers, their parents influence teenagers' behavior (Yao & Rhodes, 2015). Parents have different expectations for boys and girls, which can affect their support for their children's PA (Wigfield & Eccles, 2000). Previous studies in China have found that girls received less parental support, which had a negative impact on their exercise behavior (Hong et al., 2020).

The reason for the phenomenon that adolescents' PA declines substantially with their age may be academic pressure (Zhu et al., 2019). Junior high school students are facing greater academic pressure so they may have less time for PA. Another potential reason is parental influence. The influence of parents is important in children and adolescents' participation in sports (Hong et al., 2020). An early study found that younger children presented higher parental support from their parents and reported more physical activity than older children (Rhodes et al., 2019).

Relationship between Optimistic Latent Profiles and Physical Activity

In addition, the ANOVA results indicated that the subgroup with higher scores participated in PA more frequently, which was consistent with the SDT and previous research, which have shown a positive relationship between self-determined motivation and PA among adolescents.

The results of latent profile analysis and ANOVA have shown that compared with other profiles, the motivation and PA of Profile 1 (Low Motivation Group) were the lowest. This disparity may lead to "health inequality" phenomena and affect students'

health negatively in this subgroup, although this subgroup had the fewest students. In particular, the score of amotivation was relatively higher than any other dimension in this subgroup. Additionally, the score of intrinsic motivation and external motivation was lower than Profile 2 and Profile 3. Amotivation is the result of people feeling that doing something is meaningless or not valuable (R. M. Ryan, 1995), and people with amotivation may not have the willingness to exercise (R. Ryan & Deci, 2000a). The previous study pointed out that amotivation had been correlated with dropout among athletes and low engagement of students in PA (Vlachopoulos & Gigoudi, 2008). Another recent study found that amotivation was associated with lower self-reported MVPA (Chapman et al., 2024). Therefore, it is necessary to decrease amotivation by raising their awareness of PA and increasing their ability beliefs (Vlachopoulos & Gigoudi, 2008).

Additionally, the PA of Profile 2 (Moderate Motivation Group) ranked second among the three subgroups and had the largest number of students. The score of amotivation was the highest compared with others' profiles, which could negatively impact a student’s PA. However, the scores of intrinsic motivation and external motivation were relatively higher than amotivation and intrinsic motivation was higher than external motivation. A previous study found that students’ intrinsic motivation positively related to PA when external motivation was at a moderate level (Phillips & Johnson, 2018). In addition, external motivation can make people feel regulated but if people agree with external viewpoints or values, external motivation can also promote their behavior (Ntoumanis et al., 2021). Therefore, intervention for

this subgroup should focus on amotivation and try to raise their awareness of PA, which makes them understand the positive value and significance of physical exercise.

Finally, the scores of intrinsic motivation and external motivation of Profile 3 (High Motivation Group) were the highest while the score of amotivation was only lower than Profile 2. Furthermore, the intrinsic motivation score was higher than external motivation in Profile 3. This result is similar to previous research that intrinsic motivation and external motivation could positively affect students' PA (Nogg et al., 2021). However, some studies found that student's intrinsic motivation was positively associated with PA whereas external motivation could not predict PA among adolescents (Esmaeilzadeh et al., 2022; Sebire et al., 2013). The reason for this difference may be that the influence of intrinsic motivation is more important than external motivation (K. Li et al., 2014), and autonomy is crucial in PA among adolescents (Dishman et al., 2015), so this subgroup has a positive attitude and intention toward PA.

Practical suggestions

This study provides a novel sight for identifying latent categories of PA and offers some practical suggestions for improving PA among adolescents.

Firstly, appropriate physical activity has a positive effect on the healthy growth of children and adolescents. Therefore, the government or schools can set up training courses for parents to raise their awareness of the value of PA and increase their

support for their children (Bassett-Gunter et al., 2017; H. Brown et al., 2016).

Secondly, the role of the school is also important in PA among adolescents. Early studies have shown that school PA support was significantly associated with student's participation in PA (Wang et al., 2017). Therefore, principals and teachers should try to provide more opportunities and a better environment to increase student's engagement in sports (Eather et al., 2023; Somerset & Hoare, 2018).

Finally, intrinsic motivation is often linked with positive outcomes, including higher levels of enjoyment, satisfaction, and long-term commitment to sports (R. Ryan & Deci, 2000b). Therefore, physical education teachers can appropriately reduce the difficulty of sports rules and increase games in physical education, thereby increasing their enjoyment and flow (Burton et al., 2011; Elliott et al., 2020), which can promote students' sports engagement and decrease the drop out of students.

Limitations and shortcomings

The current study is limited by some shortcomings. Firstly, although IPAQ-SF is considered an effective method to assess physical activity, the result measured by this tool may be higher or lower (Lee et al., 2011). Therefore, future research can use some objective methods, such as an accelerometer, to measure students' physical activity. Secondly, this study only investigated adolescents in the Chinese Mainland and did not cover Western samples. Expanding studies should include students from other countries which can provide a more comprehensive view of the mechanism of adolescent participation in PA. Finally, the result from LPA may be affected by

external factors (such as society and environment) so further research should examine the stability of motivational profiles.

Conclusion

In sum, the diversity of adolescent sports motivation types reflects individual differences and also suggests the need for more personalized strategies to promote adolescent participation in sports activities. The results of this study suggested that the motivation of PA could be divided into three subgroups, and adolescents with higher intrinsic and external motivation were more active. In addition, future sports interventions need to pay attention to the phenomenon of low participation among students with low motivation, girls and older adolescents. This study offers a novel sight for adolescents' PA by latent profile analysis, which is valuable for informing intervention strategies for inactive adolescents.

Reference

Antunes, H., Rodrigues, A., Sabino, B., Alves, R., Correia, A., & Lopes, H. (2024). The effect of motivation on physical activity among middle and high school students. *Sports*, *12*(6). https://doi.org/10.3390/sports12060154

Bandura, A. (1977). Self-efficacy: Toward a unifying theory of behavioral change. *Psychological Review*.

Bandura, A. (1997). *Self-efficacy: The exercise of control*. Macmillan.

Bassett-Gunter, R., Rhodes, R., Sweet, S., Tristani, L., & Soltani, Y. (2017). Parent support for

children's physical activity: A qualitative investigation of barriers and strategies. *Research Quarterly for Exercise and Sport*, *88*(3), 282–292.

Biddle, S. J., Atkin, A. J., Cavill, N., & Foster, C. (2011). Correlates of physical activity in youth: A review of quantitative systematic reviews. *International Review of Sport and Exercise Psychology*, *4*(1), 25–49.

Brown, D., Dudley, D., & Cairney, J. (2020). Physical literacy profiles are associated with differences in children's physical activity participation: A latent profile analysis approach. *JOURNAL OF SCIENCE AND MEDICINE IN SPORT*, *23*(11), 1062–1067. https://doi.org/10.1016/j.jsams.2020.05.007

Brown, H., Atkin, A., Panter, J., Wong, G., Chinapaw, M. J., & Van Sluijs, E. (2016). Family-based interventions to increase physical activity in children: A systematic review, meta-analysis and realist synthesis. *Obesity Reviews*, *17*(4), 345–360.

Burke, S., Sharp, L., Woods, D., & Paradis, K. (2024). Enhancing parental support through parent-education programs in youth sport: A systematic review. *INTERNATIONAL REVIEW OF SPORT AND EXERCISE PSYCHOLOGY*, *17*(1), 208–235. https://doi.org/10.1080/1750984X.2021.1992793

Burton, D., Gillham, A., & Hammermeister, J. (2011). Competitive engineering: Structural climate modifications to enhance youth athletes' competitive experience. *International Journal of Sports Science & Coaching*, *6*(2), 201–217. https://doi.org/10.1260/1747-9541.6.2.201

Chalabaev, A., Sarrazin, P., Fontayne, P., Boiché, J., & Clément-Guillotin, C. (2013). The influence of sex stereotypes and gender roles on participation and performance in sport and exercise: Review and future directions. *Psychology of Sport and Exercise*, *14*(2),

136–144.

Chapman, J., Korman, N., Malacova, E., Robertson, C., Arnautovska, U., Siskind, D., Suetani, S., Stubbs, B., & Vancampfort, D. (2024). Which behavioral regulations predict physical activity and sedentary behavior in people with mental illness? *Psychological Medicine*. https://doi.org/10.1017/S0033291724001879

Chaput, J., Willumsen, J., Bull, F., Chou, R., Ekelund, U., Firth, J., Jago, R., Ortega, F., & Katzmarzyk, P. (2020). 2020 WHO guidelines on physical activity and sedentary behaviour for children and adolescents aged 5-17years: Summary of the evidence. *International Journal of Behavioral Nutrition and Physical Activity*, *17*(1). https://doi.org/10.1186/s12966-020-01037-z

Chen, H., Chen, H., Lu, T., Liu, C., Hu, C., Wang, C., & Yan, J. (2024). Personality profiles and physical activity across adolescent: Based on latent profile analysis. *Acta Psychologica*, *249*. https://doi.org/10.1016/j.actpsy.2024.104455

Chen, H., Sun, H., & Dai, J. (2017). Peer support and adolescents' physical activity: The mediating roles of self-efficacy and enjoyment. *Journal of Pediatric Psychology*, *42*(5), 569–577. https://doi.org/10.1093/jpepsy/jsw103

Collins, L. M., & Lanza, S. T. (2009). *Latent class and latent transition analysis: With applications in the social, behavioral, and health sciences* (Vol. 718). John Wiley & Sons.

Craig, C., Marshall, A., Sjöström, M., Bauman, A., Booth, M., Ainsworth, B., Pratt, M., Ekelund, U., Yngve, A., Sallis, J., & Oja, P. (2003). International physical activity questionnaire: 12-country reliability and validity. *Medicine & Science in Sports & Exercise*, *35*(8), 1381–1395. https://doi.org/10.1249/01.MSS.0000078924.61453.FB

Crane, J., & Temple, V. (2015). A systematic review of dropout from organized sport among children and youth. *European Physical Education Review*, *21*(1), 114–131. https://doi.org/10.1177/1356336X14555294

Davies, P., Spencer, S., Quinn, D., & Gerhardstein, R. (2002). Consuming images: How television commercials that elicit stereotype threat can restrain women academically and professionally. *Personality and Social Psychology Bulletin*, *28*(12), 1615–1628. https://doi.org/10.1177/014616702237644

Dishman, R., Mciver, K., Dowda, M., Saunders, R., & Pate, R. (2015). Motivation and behavioral regulation of physical activity in middle school students. *Medicine & Science in Sports & Exercise*, *47*(9), 1913–1921. https://doi.org/10.1249/MSS.0000000000000616

Doggui, R., Gallant, F., & Bélanger, M. (2021). Parental control and support for physical activity predict adolescents' moderate to vigorous physical activity over five years. *International Journal of Behavioral Nutrition and Physical Activity*, *18*(1). https://doi.org/10.1186/s12966-021-01107-w

Dominick, G., Dunsiger, S., Pekmezi, D., & Marcus, B. (2013). Health literacy predicts change in physical activity self-efficacy among sedentary latinas. *Journal of Immigrant and Minority Health*, *15*(3), 533–539. https://doi.org/10.1007/s10903-012-9666-7

Eagly, A. H. (1995). The science and politics of comparing women and men. *American Psychologist*, *50*(3), 145.

Eather, N., Wade, L., Pankowiak, A., & Eime, R. (2023). The impact of sports participation on mental health and social outcomes in adults: A systematic review and the 'mental health through sport' conceptual model. *Systematic Reviews*, *12*(1).

https://doi.org/10.1186/s13643-023-02264-8

Eccles, J. S. (1999). The development of children ages 6 to 14. *The Future of Children*, 30–44.

Elliott, S., Bevan, N., & Litchfield, C. (2020). Parents, girls' and australian football: A constructivist grounded theory for attracting and retaining participation. *Qualitative Research in Sport, Exercise and Health*, *12*(3), 392–413. https://doi.org/10.1080/2159676X.2019.1602560

Esmaeilzadeh, S., Rodriquez-Negro, J., & Pesola, A. J. (2022). A greater intrinsic, but not external, motivation toward physical activity is associated with a lower sitting time. *Frontiers in Psychology*, *13*, 888758.

Fan, M., Lyu, J., & He, P. (2014). Chinese guidelines for data processing and analysis concerning the international physical activity questionnaire. *Zhonghua Liu Xing Bing Xue Za Zhi= Zhonghua Liuxingbingxue Zazhi*, *35*(8), 961–964.

Ferguson, S., Moore, E., & Hull, D. (2020). Finding latent groups in observed data: A primer on latent profile analysis in mplus for applied researchers. *International Journal of Behavioral Development*, *44*(5), 458–468. https://doi.org/10.1177/0165025419881721

Gao, G., Liu, J., Xu, M., Xia, R., & Zhao, L. (2024). A historical review of promotions of physical activity for adolescents in China from 1949 to 2020. *FRONTIERS IN PUBLIC HEALTH*, *12*. https://doi.org/10.3389/fpubh.2024.1415513

Gómez-López, M., Courel-Ibáñez, J., & Granero-Gallegos, A. (2021). Profiles of motivation, fear of failure and anxiety in young handball players: A cross-sectional study. *International Journal Of Sports Science & Coaching*, *16*(3), 658–669. https://doi.org/10.1177/1747954120975107

Guthold, R., Cowan, M., Autenrieth, C., Kann, L., & Riley, L. (2010). Physical activity and sedentary behavior among schoolchildren: A 34-country comparison. *Journal of Pediatrics*, *157*(1), 43-U84. https://doi.org/10.1016/j.jpeds.2010.01.019

Haerens, L., Kirk, D., Cardon, G., De Bourdeaudhuij, I., & Vansteenkiste, M. (2010). Motivational profiles for secondary school physical education and its relationship to the adoption of a physically active lifestyle among university students. *European Physical Education Review*, *16*(2), 117–139. https://doi.org/10.1177/1356336X10381304

Hong, J., Chen, S., Tang, Y., Cao, Z., Zhuang, J., Zhu, Z., Chen, P., & Liu, Y. (2020). Associations between various kinds of parental support and physical activity among children and adolescents in shanghai, China: Gender and age differences. *BMC Public Health*, *20*(1), 1–9. https://doi.org/10.1186/s12889-020-09254-8

Howard, M., & Hoffman, M. (2018). Variable-centered, person-centered, and person-specific approaches: Where theory meets the method. *Organizational Research Methods*, *21*(4), 846–876. https://doi.org/10.1177/1094428117744021

Jago, R., Salway, R., House, D., Beets, M., Lubans, D., Woods, C., & de Vocht, F. (2023). Rethinking children's physical activity interventions at school: A new context-specific approach. *FRONTIERS IN PUBLIC HEALTH*, *11*. https://doi.org/10.3389/fpubh.2023.1149883

Kim, J. (2019). Multicollinearity and misleading statistical results. *Korean Journal Of Anesthesiology*, *72*(6), 558–569. https://doi.org/10.4097/kja.19087

Krosnick, J. A. (1999). Survey research. In J. T. Spence (Ed.), *Annual Review of Psychology* (BCI:BCI199900144506; Vol. 50, pp. 537–567).

Lee, P., Macfarlane, D., Lam, T., & Stewart, S. (2011). Validity of the international physical activity questionnaire short form (IPAQ-SF): A systematic review. *International Journal of Behavioral Nutrition and Physical Activity*, *8*. https://doi.org/10.1186/1479-5868-8-115

Li, F., & Harmer, P. (1996). Testing the simplex assumption underlying the sport motivation scale: A structural equation modeling analysis. *Research Quarterly for Exercise and Sport*, *67*(4), 396–405. https://doi.org/10.1080/02701367.1996.10607971

Li, K., Iannotti, R., Haynie, D., Perlus, J., & Simons-Morton, B. (2014). Motivation and planning as mediators of the relation between social support and physical activity among U. S. adolescents: A nationally representative study. *International Journal of Behavioral Nutrition and Physical Activity*, *11*. https://doi.org/10.1186/1479-5868-11-42

Lindwall, M., Ivarsson, A., Weman-Josefsson, K., Jonsson, L., Ntoumanis, N., Patrick, H., Thogersen-Ntoumani, C., Markland, D., & Teixeira, P. (2017). Stirring the motivational soup: Within-person latent profiles of motivation in exercise. *International Journal of Behavioral Nutrition and Physical Activity*, *14*. https://doi.org/10.1186/s12966-017-0464-4

Liu, L., Liu, Y., Zhang, T., & Luo, J. (2024). Study on the influence of levels of physical activity and socio-economic conditions on body mass index of adolescents. *International HEALTH*. https://doi.org/10.1093/inthealth/ihae083

Liu, Y., Ge, X., Li, H., Zhang, E., Hu, F., Cai, Y., & Xiang, M. (2023). Physical activity maintenance and increase in Chinese children and adolescents: The role of intrinsic motivation and parental support. *Frontiers in Public Health*, *11*, 1175439. https://doi.org/10.3389/fpubh.2023.1175439

Liu, Y., Tang, Y., Cao, Z., Chen, P., Zhang, J., Zhu, Z., Zhuang, J., Yang, Y., & Hu, Y. (2016). Results from shanghai's (China) 2016 report card on physical activity for children and youth. *Journal Of Physical Activity & HEALTH*, *13*(11), S124–S128. https://doi.org/10.1123/jpah.2016-0362

Lu, C., Stolk, R. P., Sauer, P. J., Sijtsma, A., Wiersma, R., Huang, G., & Corpeleijn, E. (2017). Factors of physical activity among Chinese children and adolescents: A systematic review. *International Journal of Behavioral Nutrition and Physical Activity*, *14*, 1–10.

Mayorga-Vega, D., Saldías, M., & Viciana, J. (2019). Physical fitness, physical activity, sedentary behavior and psychological predictors in chilean adolescents: Differences by gender. *Cultura Ciencia Y Deporte*, *14*(42), 233–241. https://doi.org/10.12800/ccd.v14i42.1337

Morin, A. J., Gagne, M., & Bujacz, A. (2016). Feature topic: Person-centered methodologies in the organizational sciences. *Organizational Research Methods*, 8–9.

Nogg, K. A., Vaughn, A. A., Levy, S. S., & Blashill, A. J. (2021). Motivation for physical activity among US adolescents: A self-determination theory perspective. *Annals of Behavioral Medicine*, *55*(2), 133–143.

Ntoumanis, N., Ng, J. Y., Prestwich, A., Quested, E., Hancox, J. E., Thøgersen-Ntoumani, C., Deci, E. L., Ryan, R. M., Lonsdale, C., & Williams, G. C. (2021). A meta-analysis of self-determination theory-informed intervention studies in the health domain: Effects on motivation, health behavior, physical, and psychological health. *Health Psychology Review*, *15*(2), 214–244.

Osborne, J. W. (2012). *Best practices in data cleaning: A complete guide to everything you need to do before and after collecting your data*. Sage publications.

Owen, K., Smith, J., Lubans, D., Ng, J., & Lonsdale, C. (2014). Self-determined motivation and physical activity in children and adolescents: A systematic review and meta-analysis. *Preventive Medicine*, *67*, 270–279. https://doi.org/10.1016/j.ypmed.2014.07.033

Paradis, K., Cooke, L., Martin, L., & Hall, C. (2013). Too much of a good thing? Examining the relationship between passion for exercise and exercise dependence. *Psychology of Sport and Exercise*, *14*(4), 493–500. https://doi.org/10.1016/j.psychsport.2013.02.003

Pelletier, L., Tuson, K., Fortier, M., Vallerand, R., Briere, N., & Blais, M. (1995). Toward a new measure of intrinsic motivation, extrinsic motivation, and amotivation in sports—The sport motivation scale (sms). *JOURNAL OF SPORT & EXERCISE PSYCHOLOGY*, *17*(1), 35–53. https://doi.org/10.1123/jsep.17.1.35

Phillips, L. A., & Johnson, M. A. (2018). Interdependent effects of autonomous and controlled regulation on exercise behavior. *Personality and Social Psychology Bulletin*, *44*(1), 49–62.

Pluta, B., Korcz, A., Krzysztoszek, J., Bronikowski, M., & Bronikowska, M. (2020). Associations between adolescents' physical activity behavior and their perceptions of parental, peer and teacher support. *Archives of Public Health*, *78*(1), 106.

Podsakoff, P. M., Podsakoff, N. P., Williams, L. J., Huang, C., & Yang, J. (2024). Common method bias: It's bad, it's complex, it's widespread, and it's not easy to fix. *Annual Review of Organizational Psychology and Organizational Behavior*, *11*(1), 17–61.

Podsakoff, P., MacKenzie, S., Lee, J., & Podsakoff, N. (2003). Common method biases in behavioral research: A critical review of the literature and recommended remedies. *JOURNAL OF APPLIED PSYCHOLOGY*, *88*(5), 879–903.

https://doi.org/10.1037/0021-9010.88.5.879

Qu, Y. (2023). Stereotypes of adolescence: Cultural differences, consequences, and intervention. *Child Development Perspectives*, *17*(3–4), 136–141. https://doi.org/10.1111/cdep.12489

Qurban, H., Wang, J., Siddique, H., Morris, T., & Qiao, Z. (2019). The mediating role of parental support: The relation between sports participation, self-esteem, and motivation for sports among Chinese students. *Current Psychology*, *38*, 308–319.

Rhodes, R. E., Spence, J. C., Berry, T., Faulkner, G., Latimer-Cheung, A. E., O'Reilly, N., Tremblay, M. S., & Vanderloo, L. (2019). Parental support of the canadian 24-hour movement guidelines for children and youth: Prevalence and correlates. *BMC Public Health*, *19*, 1–12.

Rodrigues, F., & Teixeira, D. (2023). The relationships between passions, intentions, habit and exercise frequency. *Journal of Sports Sciences*, *41*(8), 803–811. https://doi.org/10.1080/02640414.2023.2240622

Rodríguez-Muñoz, S., Corella, C., Abarca-Sos, A., & Zaragoza, J. (2017). Validation of three short physical activity questionnaires with accelerometers among university students in spain. *JOURNAL OF SPORTS MEDICINE AND PHYSICAL FITNESS*, *57*(12), 1660–1668. https://doi.org/10.23736/S0022-4707.17.06665-8

Ryan, R., & Deci, E. (2000a). Intrinsic and extrinsic motivations: Classic definitions and new directions. *Contemporary Educational Psychology*, *25*(1), 54–67. https://doi.org/10.1006/ceps.1999.1020

Ryan, R., & Deci, E. (2000b). Self-determination theory and the facilitation of intrinsic motivation, social development, and well-being. *American Psychologist*, *55*(1), 68–78.

https://doi.org/10.1037/0003-066X.55.1.68

Ryan, R. M. (1995). Psychological needs and the facilitation of integrative processes. *Journal of Personality*, *63*(3), 397–427.

Satorra, A. (1994). Corrections to test statistics and standard errors on covariance structure analysis. *Latent Variable Analysis/Sage*.

Sebire, S., Jago, R., Fox, K., Edwards, M., & Thompson, J. (2013). Testing a self-determination theory model of children's physical activity motivation: A cross-sectional study. *International Journal of Behavioral Nutrition and Physical Activity*, *10*. https://doi.org/10.1186/1479-5868-10-111

Slater, A., & Tiggemann, M. (2011). Gender differences in adolescent sport participation, teasing, self-objectification and body image concerns. *Journal of Adolescence*, *34*(3), 455–463. https://doi.org/10.1016/j.adolescence.2010.06.007

Somerset, S., & Hoare, D. J. (2018). Barriers to voluntary participation in sport for children: A systematic review. *BMC Pediatrics*, *18*, 1–19.

Spurk, D., Hirschi, A., Wang, M., Valero, D., & Kauffeld, S. (2020). Latent profile analysis: A review and 'how to' guide of its application within vocational behavior research. *JOURNAL OF VOCATIONAL BEHAVIOR*, *120*. https://doi.org/10.1016/j.jvb.2020.103445

Sterdt, E., Liersch, S., & Walter, U. (2014). Correlates of physical activity of children and adolescents: A systematic review of reviews. *Health Education Journal*, *73*(1), 72–89.

Tein, J., Coxe, S., & Cham, H. (2013). Statistical power to detect the correct number of classes in latent profile analysis. *Structural Equation Modeling-a Multidisciplinary Journal*, *20*(4),

640–657. https://doi.org/10.1080/10705511.2013.824781

Terwee, C., Mokkink, L., van Poppel, M., Chinapaw, M., van Mechelen, W., & de Vet, H. (2010). Qualitative attributes and measurement properties of physical activity questionnaires a checklist. *SPORTS MEDICINE*, *40*(7), 525–537. https://doi.org/10.2165/11531370-000000000-00000

van Sluijs, E., Ekelund, U., Crochemore-Silva, I., Guthold, R., Ha, A., Lubans, D., Oyeyemi, A., Ding, D., & Katzmarzyk, P. (2021). Physical activity behaviours in adolescence: Current evidence and opportunities for intervention. *Lancet*, *398*(10298), 429–442. https://doi.org/10.1016/S0140-6736(21)01259-9

Vlachopoulos, S., & Gigoudi, M. (2008). Why don't you exercise? Development of the amotivation toward exercise scale among older inactive individuals. *JOURNAL OF AGING AND PHYSICAL ACTIVITY*, *16*(3), 316–341. https://doi.org/10.1123/japa.16.3.316

Vu, M. B., Murrie, D., Gonzalez, V., & Jobe, J. B. (2006). Listening to girls and boys talk about girls' physical activity behaviors. *Health Education and Behavior*, *33*(1), 81–96.

Wang, L., Tang, Y., & Luo, J. (2017). School and community physical activity characteristics and moderate-to-vigorous physical activity among Chinese school-aged children: A multilevel path model analysis. *Journal of Sport and Health Science*, *6*(4), 416–422.

Warburton, D., & Bredin, S. (2017). Health benefits of physical activity: A systematic review of current systematic reviews. *Current Opinion in Cardiology*, *32*(5), 541–556. https://doi.org/10.1097/HCO.0000000000000437

White, R., Babic, M., Parker, P., Lubans, D., Astell-Burt, T., & Lonsdale, C. (2017).

Domain-specific physical activity and mental health: A meta-analysis. *American Journal of Preventive Medicine*, *52*(5), 653–666. https://doi.org/10.1016/j.amepre.2016.12.008

Wigfield, A., & Eccles, J. S. (2000). Expectancy–value theory of achievement motivation. *Contemporary Educational Psychology*, *25*(1), 68–81.

Williams, D. M., & Rhodes, R. E. (2016). The confounded self-efficacy construct: Conceptual analysis and recommendations for future research. *Health Psychology Review*, *10*(2), 113–128. https://doi.org/10.1080/17437199.2014.941998

Xiang, C., Zhao, J., Tengku Kamalden, T. F., Dong, W., Luo, H., & Ismail, N. (2023). The effectiveness of child and adolescent sports engagement in China: An analysis of china's results for the 2016–2022 global matrix report cards on physical activity. *Humanities and Social Sciences Communications*, *10*(1), 1–12.

Yan, W., Wang, Y., Yuan, Y., Farid, M., Zhang, P., & Peng, K. (2024). Timing matters: A longitudinal study examining the effects of physical activity intensity and timing on adolescents' mental health outcomes. *Journal of Youth and Adolescence*, *53*(10), 2320–2331. https://doi.org/10.1007/s10964-024-02011-9

Yao, C., & Rhodes, R. (2015). Parental correlates in child and adolescent physical activity: A meta-analysis. *INTERNATIONAL JOURNAL OF BEHAVIORAL NUTRITION AND PHYSICAL ACTIVITY*, *12*. https://doi.org/10.1186/s12966-015-0163-y

Zhu, Z., Tang, Y., Zhuang, J., Liu, Y., Wu, X., Cai, Y., Wang, L., Cao, Z., & Chen, P. (2019). Physical activity, screen viewing time, and overweight/obesity among Chinese children and adolescents: An update from the 2017 physical activity and fitness in Chinathe youth study. *BMC Public Health*, *19*. https://doi.org/10.1186/s12889-019-6515-9

Zhu, Z., Yang, Y., Kong, Z., Zhang, Y., & Zhuang, J. (2017). Prevalence of physical fitness in Chinese school-aged children: Findings from the 2016 physical activity and fitness in China—The youth study. *Journal of Sport and Health Science*, *6*(4), 395–403.

Click here to access/download;Figure;Figure 1.tif

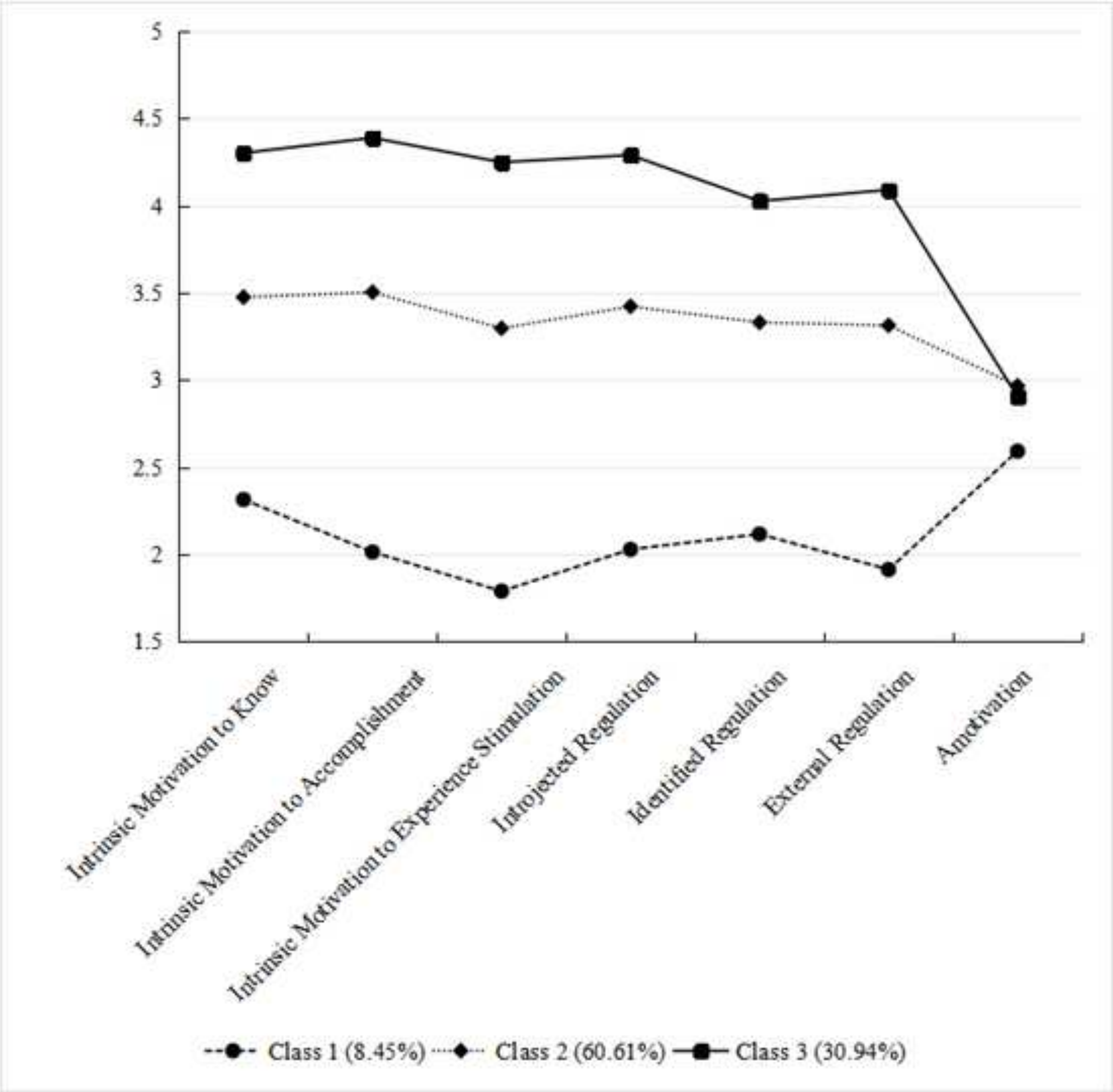

Supplement: Attachment — Submitted filename: PONE-D-25-04270 (2).pdf [file pone.0328383.s001.pdf]
