# Supplementary material for: Motivation and physical activity across Chinese adolescents: Based on latent profile analysis
Source: PLoS One. 2025 Jul 30;20(7):e0328383. doi: 10.1371/journal.pone.0328383 (PMC12310012; doi:10.1371/journal.pone.0328383)
Supplement: Attachment — Submitted filename: Response to Reviewer.docx [file pone.0328383.s003.docx]

Dear Reviewer,

Thank you very much for your time and valuable feedback on our manuscript. We sincerely appreciate your constructive comments, which have helped us improve the quality and clarity of our work. Below, we provide a point-by-point response to your suggestions and outline the revisions we have made to the manuscript.

1. Abstract Rewriting

We have revised the Method section of the abstract to provide a clearer and more detailed explanation of the methodology. The updated version now includes key aspects such as the sample size, measurement tools (Sport Motivation Scale and IPAQ-SF), and analytical methods (LPA, MANOVA, ANOVA). This revision ensures that the abstract better reflects the study's design and procedures.

2. In-text Citation Format

We have carefully checked and corrected all in-text citations throughout the manuscript to ensure they adhere to the required format (last name and year only). Any deviations have been rectified.

3. Clarification of Research Gap

As suggested, we have expanded the discussion of the research gap in the Introduction section (page 7-8 of the revised manuscript). Specifically, we highlighted the lack of person-centered studies on motivation profiles among Chinese adolescents and the unique sociocultural context that may influence these profiles. This addition underscores the novelty and significance of our study.

4. Rationale for Using SMS Instead of SMS-2

We acknowledge the availability of the newer SMS-2 scale and appreciate the opportunity to clarify our choice. Our decision to use the original SMS was based on the following considerations:

**Factor Structure:** The SMS has demonstrated robust structural validity in prior studies, including those involving Chinese adolescents.

**Internal Consistency**: The scale’s reliability (Cronbach’s α) for all subscales in our study was acceptable (α > 0.70).

**Dimensionality**: The SMS-2 consolidates external motivation into a single dimension, whereas the SMS allows for a finer-grained analysis of introjection, identification, and external regulation-critical for our research questions.

6. Model Fit Indices (AIC, BIC, aBIC)

We have supplemented the explanation of model fit indices in the Results section (page 11). Specifically, we noted that differences >10 in AIC and >6 in BIC/aBIC are considered meaningful for model selection (Spurk et al., 2020; Tein et al., 2013). This addition clarifies the criteria used to determine the optimal profile model.

7. Description of Amotivation Correlations

We have revised the paragraph discussing amotivation correlations (page 13) to ensure accuracy and clarity. The updated text now clearly states that amotivation was not significantly correlated with intrinsic motivation to accomplishment or experience stimulation but showed weak positive correlations with identification and external regulation.

8. Conclusion Wording

The phrase “In sum” has been replaced with “In summary” in the Conclusion section.

9. Proofreading

The manuscript has undergone rigorous proofreading to correct grammatical errors, improve sentence structure, and ensure consistency in terminology. We have also double-checked tables and figures for accuracy.

Once again, we deeply appreciate your thoughtful review and hope that our revisions have addressed all your concerns satisfactorily. Please do not hesitate to contact us if further clarifications or adjustments are needed.

Best regards,

Wei Zheng

Sanming University

Email: zhengweiSanmingUniversity@outlook.com
